# Fe/Mn Multilayer Nanowires as High-Performance T_1_-T_2_ Dual Modal MRI Contrast Agents

**DOI:** 10.3390/ma14092238

**Published:** 2021-04-27

**Authors:** Xiaoming Cao, Liyuan Gu, Shike Hu, Aiman Mukhtar, Kaiming Wu

**Affiliations:** 1 The State Key Laboratory of Refractories and Metallurgy, Hubei Province Key Laboratory of Systems Science in Metallurgical Process, International Research Institute for Steel Technology, Collaborative Innovation Center for Advanced Steels, Wuhan University of Science and Technology, Wuhan 430081, China; xmcao508@126.com; 2 Research and Development Center for Non-Powered Nuclear Technology, Hubei University of Science and Technology, Xianning 437000, China; gly0726@126.com (L.G.); 18771287873@126.com (S.H.)

**Keywords:** dual modal, MRI, CAs, nanowire, relaxation

## Abstract

A lot of nanomaterials are using T_1_-T_2_ dual mode magnetic resonance (MR) contrast agents (CAs), but multilayer nanowire (NW) with iron (Fe) and manganese (Mn) as T_1_-T_2_ dual modal CAs has not been reported yet. Herein, we synthesized a Fe/Mn multilayer NW with an adjustable Fe layer, as T_1_-T_2_ dual-mode CAs. The relaxation performance of Fe/Mn multilayer NW was studied at 1.5 T. Results show that, when the length of the Fe layer is about 10 nm and the Mn is about 5 nm, the r_1_ value (21.8 mM^−1^s^−1^) and r_2_ value (74.8 mM^−1^s^−1^) of the Fe/Mn multilayer NW are higher than that of Mn NW (3.7 mM^−1^s^−1^) and Fe NW (59.3 mM^−1^s^−1^), respectively. We predict that our Fe/Mn multilayer NW could be used as T_1_-T_2_ dual mode MRI CAs in the near future.

## 1. Introduction

Magnetic resonance imaging (MRI) is a very powerful technique, widely used in clinical medical imaging technology and in scientific research. It uses static magnetic fields and radiofrequency magnetic fields to image human tissues. In the imaging process, neither ionization radiation nor a contrast agent can be used to obtain clear images with high contrast. The application of MRI in medical diagnosis can be roughly summarized into seven aspects: (1) tumor diagnosis and evaluation [1], (2) vascular display [2], (3) dynamic joint observation and detection of small lesions [3], (4) diagnosis and tracking of stroke [4], (5) cardiac imaging and cardiac function assessment, (6) surgery and interventional therapy [5].

In general, the use of CAs can enhance contrast between the area of interest (RIO) and the surrounding tissues, thus improving the sensitivity of the MRI [6,7], and can interact with the surrounding water particles to shorten their relaxation time [8,9,10], thus producing brighter and darker images on T_1_ and T_2_ images [11,12,13]. After years of development, CAs are broadly divided into T_1_ CAs and T_2_ CAs. T_1_ CAs are usually paramagnetic, including gadolinium complexes, manganese salts or manganese chelates, and the Gd_3_-based complexes [14,15,16]. Manganese chelates (new dendritic DTPA manganese chelates [17], Mn-DOTA [18]) are mostly used in clinical diagnosis nowadays. T_2_ CAs are usually superparamagnetic particles and ferromagnetic particles, and the superparamagnetic iron oxide nanoparticles (SPIOS) are the most widely studied and can also be used in hyperthermia [19], in addition to being often used as T_2_ CAs [20,21]. Although the development of CAs has achieved great success, this single-mode contrast agent is facing many challenges in the new technology. Positive CAs generally have large permanent magnetic moments, can shorten T_1_ relaxation time, improving imaging sensitivity, but they are more toxic and take less time to circulate [22,23]. Negative CAs, despite their large saturation magnetization, can help distinguish between normal and diseased tissues and have a long half-life, but these effects are accomplished by reducing the imaging signal; the reduced signal intensity can lead to the darkening of the image and poor resolution, and artifacts are generated when disturbed by high magnetic field inhomogeneity and sensitivity effects. It makes the lesion easily confused with the low signal generated by near normal tissues, so the sensitivity for pathological diagnosis is slightly poor [24,25,26].

The advantage of T_1_-T_2_ dual-mode CAs over single-mode CAs is that the two CAs complement each other. Under the same metabolic conditions, T_1_ weighted imaging and T_2_ weighted imaging can be obtained for the same lesion site. Achieving accurate matching of two weighted images in time and space effectively eliminates pseudosignals and provides a higher resolution imaging effect [27,28,29]; on the other hand, compared with other kinds of dual-mode CAs, such as PET/MRI [30], PAI/MRI [31,32], and US/MRI [33], T_1_-T_2_ dual-mode CAs can simultaneously achieve the MRI-weighted imaging of two modes on the same instrument. This imaging mode can effectively avoid the different imaging images [21].

One of the direct ideas to endow CAs with the T_1_-T_2_ dual-mode angiography function is to combine T_1_ and T_2_ contrast materials. A typical method is to combine Gd^3+^ and Mn^2+^ ions with iron oxide NPs. According to this idea, researchers have devised various forms of dual-mode CAs. Li et al. [24] prepared T_1_-T_2_ dual-mode CAs with Fe_3_O_4_ as a core and Gd_2_O_3_ as a shell, which has a good imaging effect. Zhou et al. [22] uniformly doped Gd_2_O_3_ into iron oxide nanoparticles. The signal-to-noise ratio (SNR) of T_1_ and T_2_ images was improved effectively. Cheng et al. [7] combined IONP, Pt, and gold nanocrystals into dumbbells, and T_1_-T_2_ dual mode MR CAs were obtained. Gao et al. [21] prepared gadolinium–iron oxide nanoparticles via polyethyleneimine encapsulation (GdIO-stPEI).

So far, most of the studies on T_1_-T_2_ dual-mode CAs have focused on spherical zero-dimensional magnetic nanoparticles, which are mainly combined with magnetic nanoparticles with T_1_ and T_2_ imaging capabilities using chemical methods [34]. This combination method has complex steps and poses great difficulty, and the size of the synthesized products is uneven. Compared with zero-dimensional magnetic nanoparticles, one-dimensional magnetic nanomaterials are characterized by geometric anisotropy and a larger body surface area [7,34], which makes their circulation time in the blood longer and their penetration in porous tissues (tumor sites) stronger [34,35]. Magnetic fields that can induce greater inhomogeneity [36] increase the chance of nanomaterials contacting water molecules [37]. In addition, one-dimensional magnetic NWs prepared by electrodeposition using the template method have strong size control [35] and can combine T_1_ and T_2_ CA components through codeposition and multilayer structure design. The method is simple and easy to operate. Therefore, the study on the relaxation performance of one-dimensional magnetic nanomaterials is of great significance. We have prepared Fe/Mn multilayer NWs before, which used Fe components with high susceptibility as T_2_ contrast materials and unpaired electronic Mn components as T_1_ contrast materials, and studied their relaxation. We found that multilayer NWs exhibited T_1_-T_2_ dual-mode enhancement compared with single-component NWs. However, compared with T_1_ and T_2_ CAs already in commercial application, the relaxation degree of Fe/Mn multilayer NWs prepared by us is relatively low (r_1_ = 1.25 mM^−1^s^−1^ and r_2_ = 5.13 mM^−1^s^−1^) [36]. In order to improve the relaxation degree of Fe/Mn multilayer NWs, this paper adjusted the sublayer thickness of Fe/Mn multilayer NWs, analyzed the influence of the sublayer thickness of Fe/Mn multilayer NWs on the magnetic properties and relaxation degree of NWs, and evaluated the T_1_-T_2_ dual-mode imaging ability of Fe/Mn multilayer NWs.

## 2. Experimental Details

### 2.1. Materials and Characterization

Aluminum sheet (99.999%) was purchased from GRINM (Beijing, China), FeSO_4_·7H_2_O (AR, 99%), H_3_BO_3_ (AR, 99.5%), Vitamin C (AR, 99%), and MnSO_4_·H_2_O (AR, 99%) were purchased from SINOPHARM (Beijing, China). SnO_2_ (AR, 99.5%), NH_3_·H_2_O (AR, 25~28%) was purchased from Aladdin (Shanghai, China). Magnevist and Combidex were purchased from Bayer Healthcare Co., LTD. (Beijing, China). TEOS and (NH_4_)_2_SO_4_ were purchased from Sinopharm Chemical Reagent Co., Ltd. (Beijing, China). All chemicals were used as received without further purification. 

The surface structure of an aluminum anodic oxide (AAO) template with NWs was observed using a field emission scanning electron microscope (FESEM, ZEISS-Utral55, Germany). The TEM (transmission electron microscopy, JEOL-2100F, Japan) was used to study the structure of NWs. In order to release the NWs from the AAO template for TEM observation, first they were dissolved in a 5% NaOH solution, then washed with DI water many times. Finally, the NWs were dispersed in the ethanol solution. X-ray diffraction (XRD, Germany) was used to analyze the crystal structure of the NWs. To study the distribution data of the elements, EDX was done on JEOL ARM-200F FESEM (Japan), operating at 200 kV accelerating voltage. An energy dispersive spectrometer (EDS, ZEISS-Utral55) was used to study the elemental composition of NWs. The SiO_2_ coated on the surface of NWs was characterized by IR Prestige-21. The concentration of elements in the nanowire dispersion system was measured by Optima 7300 DV. The zeta potential of the nanowire dispersion system was characterized by Zetasizer (UK). The magnetic properties of NWs were analyzed by ADE Model4HF VSM under a magnetic field up to 20kOe. The T_1_ or T_2_ phantom MR images were obtained on a 1.5 T scanner (Symphony, Siemens Medical systems, Erlangen, Germany).

### 2.2. Synthesis of Fe and Mn NWs

The synthesis of an AAO template was discussed in our previously published paper [36], this process uses a two-step anodization method. AAO templates with 30 nm diameter were used to synthesize the Fe NWs. The electrochemical deposition of Fe NWs was done using an electrolytic solution of 80.4 g/L FeSO_4_·7H_2_O, 25g/L H_3_BO_3_, and 0.1 g/L ascorbic acid at −1.5 V for 36 s using the direct current deposition technique. AAO templates with 30 nm diameter were also used to synthesize the Mn NWs using electrodeposition, using direct current deposition at −2.0 V for 65 s. The composition of electrolytes was 120 g/L MnSO_4_, 130 g/L (NH_4_)_2_SO_4,_ and 1 g/LSnO_2_. The pH of the solution was adjusted to 6.0–7.5 by adding a little ammonia and sulfuric acid solution.

The three-electrode cell was used during the electrochemical deposition at room temperature. The cathode was the AAO templates, which sputtered gold layer at the back side, the anode was platinum electrode, and the reference electrode was a saturated calomel electrode.

### 2.3. Synthesis of Fe/Mn Multilayer NWs

**Figure 1:**
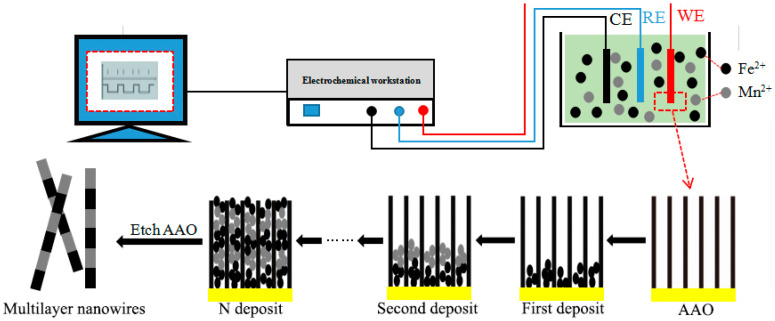
Schematic illustration of deposition Fe/Mn multilayer NWs array.

For the electrochemical deposition of Fe/Mn multilayer NWs, the composition of the electrolyte used was 1.5 g/L FeSO_4_·7H_2_O, 215 g/L MnSO_4_, 130 g/L (NH_4_)_2_SO_4_, 1 g/L SnO_2_, and 25 g/L H_3_BO_3_. The solution pH was adjusted to 6.0–7.5 by adding a little H_2_SO_4_ and NH_3_ solution. The deposition voltage, deposition time, and cyclic deposition times of Fe and Mn layers are shown in Table 1. The template was washed with deionized water and dried after deposition. The deposition process is shown in Figure 1.

### 2.4. Surface Functionalization of the NWs

Because the magnetic NWs have poor hydrophilicity, they can easily gather; hence, they can not be used directly to analyze the relaxation efficiency. So the Fe/Mn multilayer NWs were functionalized with mSiO_2_, as silica has good biocompatibility, is known to be nontoxic, harmless and hydrophilic, and has chemical, mechanical, and thermal stability [7,21,34]. The specific operation is as follows: A certain number of prepared NWs were evenly dispersed in the mixed solution of 140 mL of ethanol and 20 mL of distilled water to form a colloidal solution. After ultrasonic treatment for 30 min, the NWs were fully dispersed in the solution. Then, 2 mL of concentrated ammonia water was added into the solution. After 30 min of mechanical stirring, a mixed solution of 1.5 mL TEOS and 10 mL ethanol were added drop-wise into the colloidal solution and stirred for 3 h at room temperature. In order to remove the excess SiO_2_ impurities and the residual ammonia water, after the reaction, the solution was washed repeatedly with ethanol and distilled water until it was no longer turbid. The precipitate was dried in a vacuum drying oven for standby use. We could also coat the Fe NWs and Mn NWs with mSiO_2_ using the above method.

### 2.5. MRI in Solution

The Fe/Mn@SiO_2_ multilayer NWs sample was configured with a dispersed solution with a concentration gradient of Mn (0~0.5 mm) and a concentration gradient of Fe (0~0.5 mm), and whose longitudinal relaxation rates, 1/T_1_, and transverse relaxation rates, 1/T_2_, were measured respectively. Their relaxation degrees, r_1_ and r_2_, were obtained through the slope of the fitting line.

The actual determination of [Mn] and [Fe] are as follows, of which [Mn] of 1# sample: 0, 0.033, 0.057, 0.129, 0.245, 0.521 mm, [Fe]: 0, 0.028, 0.059, 0.121, 0.237, 0.513 mm; [Mn] of 2# sample: 0, 0.035, 0.062, 0.116, 0.247, 0.488 mM, [Fe]: 0, 0.041, 0.062, 0.118, 0.233, 0.516 mM; [Mn] of 3# sample: 0, 0.029, 0.067, 0.125, 0.261, 0.489 mM, [Fe]: 0, 0.032, 0.061, 0.106, 0.213, 0.506 mM; [Mn] of # 4 samples: 0, 0.031, 0.065, 0.118, 0.236, 0.503 mM, [Fe]: 0, 0.029, 0.059, 0.137, 0.272, 0.594 mM. The control group was treated with a Mn@SiO_2_ nanowire dispersion solution with [Mn]: 0, 0.032, 0.058, 0.112, 0.261, 0.499 mM and a Fe@SiO_2_ nanowire dispersion solution with [Fe]: 0, 0.025, 0.072, 0.121, 0.251, 0.507 mM.

## 3. Results and Discussion

### 3.1. Synthesis and Characterization

**Figure 2:**
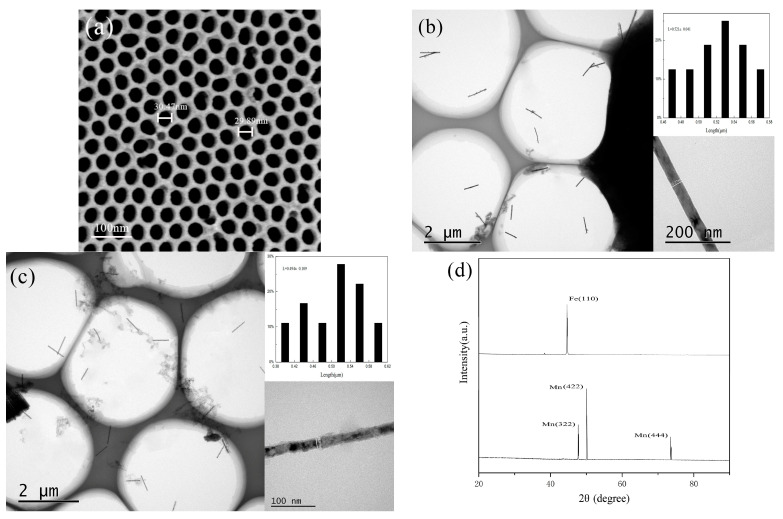
Characterization of Fe and Mn NWs: (**a**) SEM images of AAO template; (**b**) TEM image of as-deposited Fe NWs arrays; the up-right and down-right inset respectively were the size distributions and a single NW; (**c**) TEM image of as-deposited Mn NWs arrays; the up-right and down-right inset, respectively, were the size distributions and a single NW; (**d**) X-ray diffraction pattern of Fe and Mn NWs.

Figure 2a is a FESEM diagram of the front side of the AAO template prepared in this paper. It can be seen that the template holes are uniformly distributed and orderly, and that the pore diameter is about 30 nm. Figure 2b shows the TEM images of Fe NWs; it is clear from the image that the outer wall of the as-prepared NWs was smooth and that the wire diameter was uniform. In order to accurately measure the length of the NW, we did not use ultrasonic dispersion. The measured NW’s length distribution was relatively narrow. It is discernible from the inset of Figure 2b that the length of NWs was about 0.5 μm (0.521 ± 0.041 μm), and the diameter was about 30 nm (31.27 nm). A TEM diagram of Mn NWs is shown in Figure 2c. It can be seen that the morphology of Mn NWs is similar to that of Fe NWs. The length and diameter of Mn NWs was approximately 0.5 μm (0.494 ± 0.109 μm) and 30 nm (28.98 nm), respectively. Figure 2d shows the X-ray diffraction pattern of Fe NWs and Mn NWs. By comparing the diffraction peak in the figure with the Fe standard card (PDF No.06-0696) and Mn standard card (PDF No.32-0637), it can be read out that both Fe NWs and Mn NWs prepared are body-centered cubic structures.

**Figure 3:**
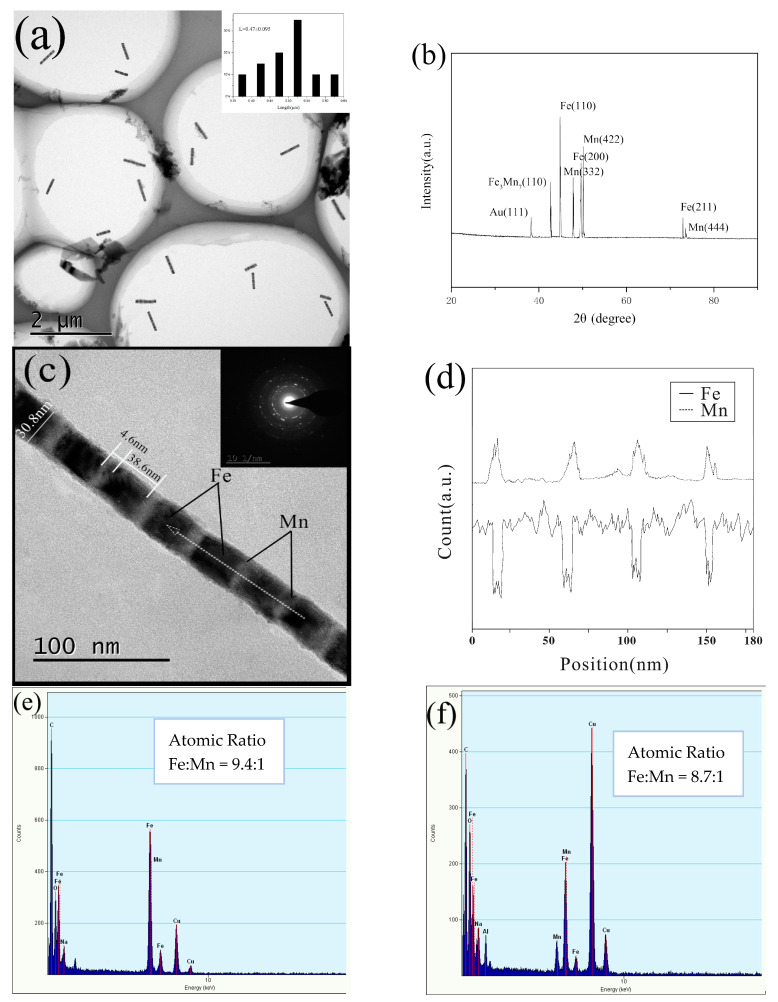
Characterization of multilayer 4# Fe/Mn NWs: (**a**) TEM image of a large number of NWs; the inset shows the length distribution of NWs; (**b**) XRD of NWs; (**c**) TEM image of a single NW; (**d**) EDX linear scan of a single NW; (**e**) EDS analysis of a single NW; (**f**) EDS spectra of a large number of NWs.

Since the morphology, structure, and composition of the prepared Fe/Mn multilayer NWs are similar, we choose 4# Fe/Mn multilayer NWs for characterization. Figure 3a shows the TEM image of multiple 4# Fe/Mn multilayer NWs, and the inset shows the size distribution. It can be seen that the Fe layer alternates with the Mn layer; the interface is clearly visible; the surface of the NW is smooth and the thickness is uniform. The length of the Fe/Mn multilayer NWs exhibited a narrow size distribution. The Fe/Mn NWs obtained alternating deposition 11 times and owned an average length of about 0.5 μm (0.47 ± 0.095 μm).

Figure 3b shows the XRD pattern of the 4# Fe/Mn multilayer nanorod array. Compared with the standard card (PDF No.06-0696, JCPDF 32-0637, PDF No.47-1409, and JCPDF 04-0784), the eight diffraction peaks in the diffraction spectrum correspond to the strong diffraction peaks of Fe (110); Mn (332), (422), (444); Fe3Mn7 (111), (200), (220); and Au (111). The results show that the phase compositions of the 4# Fe/Mn multilayer NWs are Fe and Mn of bcc structure, Au of fcc structure and Fe3Mn7 of fcc structure, respectively. The appearance of the weak Au (111) diffraction peak is mainly caused by the gold plating layer on the back of the alumina template. In addition, the existence of Fe3Mn7 indicates that codeposition of two metals occurs during the Fe, Mn alternating deposition.

A magnified individual Fe/Mn multilayer NW shown in Figure 3c indicates the diameter was about 30 nm (30.8 nm). The corresponding SAED pattern shown in the upper-right inset in Figure 3c revealed the polycrystalline structure of the Fe/Mn NWs.

Owing to the fact that the backscattering ability of Fe is greater than that of Mn, and the lining of the Fe layer is higher than the Mn layer, the darker part should be Mn with a length of about 5 nm (4.6 nm), while the brighter part corresponds to Fe with a length of about 40 nm (38.6 nm). The illustration is the electron diffraction pattern of the nanorod, indicating that both Fe and Mn layers are polycrystalline.

In order to further confirm the composition and the distribution of the elemental metal, EDX line scanning analysis was conducted on the dotted line shown in TEM Figure 3c. The result is shown in Figure 3d; the solid line represents the Fe components and the dotted line represents the Mn component. The diffraction peaks corresponding to the two components show continuous alternating on the whole, and the result was consistent with the multilayer structure observed in the TEM image in Figure 3c. Moreover, we find that, in the energy spectrum of the Mn layer, in addition to the characteristic peaks of the Mn elements, there are weak peaks of the Fe elements, but in the energy spectrum of the Fe layer, there is no corresponding characteristic peak of the Mn elements. The results indicate that the Fe layer in Fe/Mn multilayer NWs is mainly composed of Fe phase with bcc structure, while the Mn layer is composed of simple elements and the alloy Fe3Mn7 with bcc structure. The phase Fe3Mn7 occurs with the deposition of Mn components during the formation of the layer. In order to investigate the element composition of Fe/Mn multilayered NWs, we carried out an energy spectrum analysis on the same NW. The results are shown in Figure 3e. The atomic ratio of Fe to Mn is about 9.4:1. Figure 3f shows the measured metal element composition of the multiple NWs in Figure 3a by surface scanning, and the atomic ratio of Fe and Mn is about 8.7:1. The atomic ratio of Fe and Mn, measured twice, is very close, so the single Fe/Mn multilayered nanowires are representative. In addition to Fe and Mn, there are also O, Al, Cu, Na, and C in the EDS spectra. Among them, O and Al are the components of the alumina template. C comes from the C layer sprayed before the sample test, and Cu and Na come from the copper mesh and NaOH respectively.

The position of the Mn layer in Fe/Mn multilayer NWs was analyzed by energy spectrum and the results are shown in Table 2. The atomic percentage of Fe and Mn in the Mn layer is about 12.59:87.41, which means that the Mn layer is actually a Mn0.87Fe0.13 alloy layer. Although the concentration of Fe in the bath is very low, the deposition of Fe is accompanied by the deposition of the Mn layer. It is very important to control the concentration ratio of Fe and Mn in order to ensure the deposition of Fe layer and avoid the codeposition of too much Fe in the Mn layer.

**Figure 4:**
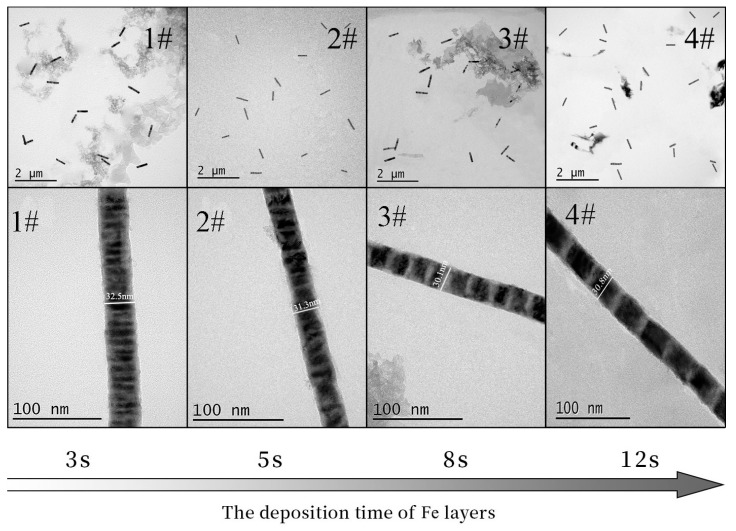
TEM images of Fe/Mn multilayer NWs with different Fe deposition times.

Figure 4 is the TEM diagram of 1#~4# Fe/Mn multilayer NWs prepared using different Fe deposition times. We can see that the four NWs are of uniform thickness and have a smooth surface, and the interface between the Mn layer and the Fe layer is clearly visible; the length of each layer is uniform. The length and diameter of the four NWs are listed in Table 3. It can be seen that the diameter (about 30 nm) and the length (about 0.5 μm) of the four NWs are relatively similar. With the increase in Fe deposition time, the length of the Fe layer increases correspondingly. The length of the Mn layer in the 1#~4# Fe/Mn multilayer NWs is about 5 nm, while the length of the Fe layer is approximately 5, 10, 20, and 40 nm, respectively. The growth rate of the Fe layer increases gradually from 2 to 3.3 nm/s, and the growth rate of the Mn layer is about 5 nm. The results show that the length of each layer can be adjusted by controlling the deposition time of each layer of the Fe/Mn multilayer NWs. Therefore, we can obtain multilayer NWs with different diameters and sublayer lengths using electrodeposition.

The length of Mn in 1# Fe/Mn multilayer NWs looks smaller than in 4# Fe/Mn multilayer NWs from TEM images in Figure 4. This may be because the Mn layer at the initial sedimentary stage is actually the competition of the Fe^2+^ and Mn^2+^ sedimentary processes before the start of the sedimentary Mn layers. 4# Fe/Mn multilayer NWs, due to the longer Fe deposits, result lower Fe^2+^ concentration in channel, compared with 1# Fe/Mn multilayer NWs, so in the initial stages of the sedimentary Mn layer 1# Fe/Mn multilayer NWs, accompanied by more codeposition of Fe^2 +^, leads to Mn layer thickness looking shorter than that of 4# Fe/Mn multilayer NWs.

### 3.2. Water-Solubilization of NWs

**Figure 5:**
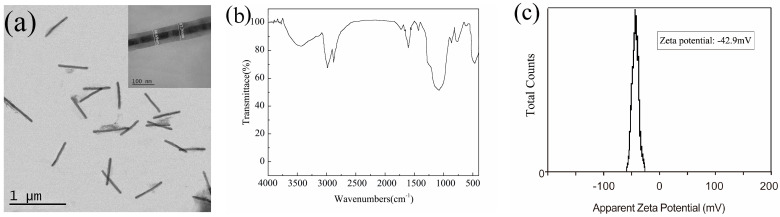
(**a**) TEM image of 4# Fe/Mn@SiO_2_ NWs; the inset shows an individual NW enlarged; (**b**) FT-IR spectra; (**c**) zeta potential.

Because the morphology and composition of NWs in this paper are similar, they have little influence on the SiO_2_ coating process, so we selected the 4# Fe/Mn multilayer NWs covered by SiO_2_ for characterization. A typical TEM image of the as-prepared Fe/Mn@SiO_2_ NWs is shown in Figure 5a. It can be seen from the figure that, after coating with SiO_2_, the NWs are relatively dispersed without a large amount of aggregation. An enlarged TEM image of an individual Fe/Mn@SiO_2_ NW in the inset indicates the core-shell structured NW has a uniform diameter of about 90 nm (86.7 nm) and a mesoprous silica layer with a thickness of 30 nm. It can be seen that the central part of each NW is darker than the outer layer, due to the fact that the atomic number of Fe or Mn is higher than that of Si, which confirms the core-shell structure of the NWs. A FT-IR spectrum analysis was performed on Fe/Mn@SiO_2_ multilayer NWs, and the results are shown in Figure 5b. The measured spectrum was completely consistent with the typical characteristic absorption bands of amorphous SiO_2_, indicating that our NWs were successfully covered by SiO_2_ [37].

The Fe/Mn multilayer NWs coated with SiO_2_ were installed into the centrifugal tube and dispersed with water. A dispersion solution with Fe concentration of about 0.521 mM was prepared. Figure 5c is the zeta potential diagram of the sample prepared. The results show that the Fe/Mn multilayer NW’s dispersion has a high zeta potential (−42.9 mV), indicating that the NWs coated with SiO_2_ have good dispersion.

### 3.3. Analysis of Magnetic Behavior

**Figure 6:**
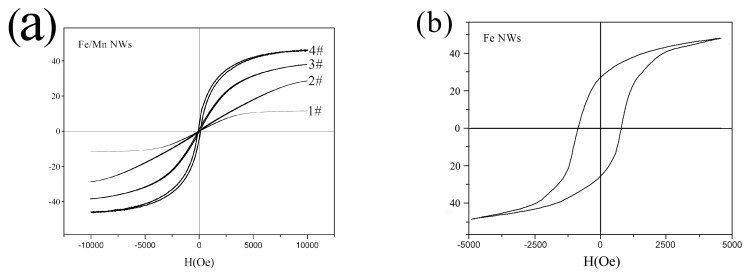
Typical hysteresis loops of NWs at 300K: (**a**) Fe/Mn@SiO_2_ multilayer NWs; (**b**) Fe@SiO_2_ NWs.

The magnetic properties of 1#~4# Fe/Mn@SiO_2_ multilayer NWs and Fe NWs were measured by VSM. The results are shown in Figure 6. The magnetic properties calculated from the hysteresis loop are listed in Table 4.

We can see in Figure 6a, as for Fe/Mn@SiO_2_ multilayer NWs, the length of the Fe layer (5 nm–40 nm) is proportional to the Hc (8.2 Oe~81.4 Oe) and Mr (0.1 emu/g~12.2 emu/g) of the Fe/Mn multilayer NWs. This may be because the Fe layer is in a single domain structure with a relatively small length, and the Hc and Mr are proportional to the size. When the length of the Fe layer is small to a certain extent, Fe/Mn multilayer NWs almost disappear and NWs show superparamagnetic characteristics, which lay a foundation for Fe/Mn multilayer NWs to become a magnetic resonance T_1_-T_2_ dual-mode contrast agent. In addition, we found that, with the decrease in the length of the Fe layer, the saturation magnetization of the Fe/Mn multilayer NWs decreased gradually (43.2~26.5 emu/g) and finally decreased significantly (11.3 emu/g), which was mainly caused by the decrease in the length of the Fe layer and the decrease of the Fe component relative to the Fe/Mn multilayer NWs. When the length of the Fe layer decreases to 5 nm, the size effect of the Fe layer becomes more obvious. At this point, the atomic structure and symmetry of the Fe layer are different from those of the internal atoms, so the saturation magnetization will be strongly reduced. As can be seen from the hysteresis cycle of Fe NWs in Figure 6b, Fe NWs exhibit ferromagnetism, and their saturation magnetization (48.1 emu/g) and coercive force (658 Oe) are significantly greater than Fe/Mn multilayer NWs, which is mainly due to the magnetic anisotropy caused by the shape anisotropy of Fe NWs.

### 3.4. The Relaxation Efficiency of Fe/Mn Multilayered NWs

To validate the potential of the Fe/Mn@SiO_2_ multilayer NWs as T_1_-T_2_ dual modal CAs, we performed their phantom MR imaging on a 1.5 T MRI scanner and calculated both their longitudinal and transverse relaxivities r_1_ and r_2_, respectively. Using self-prepared Fe NWs and Mn NWs, which were similar in size to multilayer NWs, as respective T_1_ and T_2_ controls, samples of the Fe/Mn@SiO_2_ multilayer NWs containing different metal concentrations were scanned.

**Figure 7:**
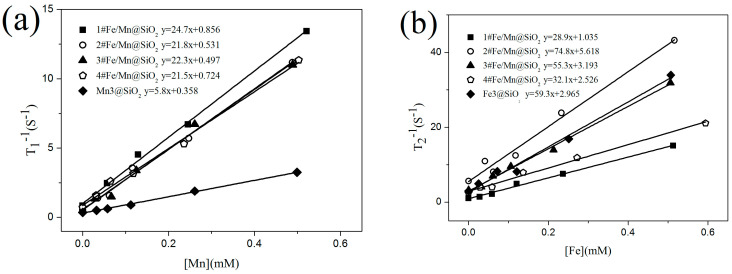

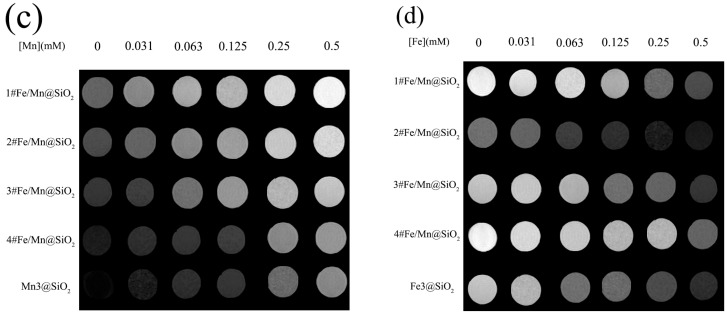
Relaxation properties of Fe/Mn@SiO_2_ NWs: (**a**) The 1#~4# Fe/Mn@SiO_2_ and Mn@SiO_2_ NWs obtained a correlation line between 1/T_1_ and Mn concentration in a 1.5T imager, and the slope of the fitting line was r_1_; (**b**) The 1#~4# Fe/Mn@SiO_2_ and Fe@SiO_2_ NWs obtained a correlation line between 1/T_2_ and Fe concentration in a 1.5T imager, and the slope of the fitting line was r_2_; (**c**) MR T_1_ weight diagram of 1#~4# Fe/Mn@SiO_2_ NWs and Mn@SiO_2_ NWs with different Mn concentrations_;_ (**d**) MR T_2_ weight diagram of 1#~4# Fe/Mn@SiO_2_ NWs and Fe@SiO_2_ NWs with different Fe concentrations.

Figure 7a,b show T_1_ and T_2_ relaxation properties of Fe/Mn@SiO_2_ multilayer NWs measured respectively, and the relevant data are listed in Table 5.

We found that the r_1_ (21.5 mM^−1^s^−1^~24.7 mM^−1^s^−1^) of 1#~4# Fe/Mn@SiO_2_ multilayer NWs was significantly higher than the r_1_ (5.7 mM^−1^s^−1^) of single-component Mn@SiO_2_ NWs with similar morphology and size, and the r_2_ (74.8 mM^−1^s^−1^) of 2# Fe/Mn@SiO_2_ multilayer NWs was higher than the r_2_ (59.5 mM^−1^s^−1^) of single-component Fe@SiO_2_ NWs with similar morphology and size. r_1_ and r_2_ of 2# Fe/Mn@SiO_2_ multilayer NWs were higher than r_1_ (3.8 mM^−1^s^−1^) and r_2_ (53 mM^−1^s^−1^) of Combidex, a T_1_ contrast agent commonly used in clinical practice [38]. In addition, the r_2_/r_1_ values of 2# Fe/Mn@SiO_2_ multilayer NWs satisfy the conditions (2 < r_2_/r_1_ < 10), indicating that 2# Fe/Mn@SiO_2_ multilayer NWs have significant T_1_-T_2_ dual-mode contrast enhancement effects. When the length of the Fe layer is 10 nm, the r_2_ (74.8 mM^−1^s^−1^) of the Fe/Mn@SiO_2_ multilayer NWs is the maximum, while, when the length of Fe layer is 5 nm, the r_1_ (24.7 mM^−1^s^−1^) of Fe/Mn@SiO_2_ multilayer NWs is the maximum.

Figure 7c,d show the T_1_- and T_2_-weighted MRI images of Fe/Mn@SiO_2_ multilayer NWs under different Mn concentration gradients and different Fe concentration gradients, respectively. As can be seen from Figure 7c, with an increase in Mn concentration, the T_1_-weighted MRI images of Fe/Mn@SiO_2_ multilayer NWs become brighter and brighter. Moreover, under the same Mn concentration, Fe/Mn@SiO_2_ multilayer NWs have a stronger contrast than Mn@SiO_2_ NWs. The contrast ratio of 1# Fe/Mn@SiO_2_ multilayer NWs is the highest, while that of 4# Fe/Mn@SiO_2_ multilayer NWs is the weakest. As can be seen from Figure 7d, with an increase in Fe concentration, the T_2_-weighted MRI image of Fe/Mn@SiO_2_ multilayer NWs becomes darker and darker. Moreover, at the same Fe concentration, the contrast ratio of 2# Fe/Mn@SiO_2_ multilayer NWs is stronger than that of Fe@SiO_2_ NWs, while the contrast ratio of 1# Fe/Mn@SiO_2_ multilayer NWs is the weakest. The above results are consistent with the relaxation data, indicating that the 2# Fe/Mn@SiO_2_ multilayer NWs have the potential to become a T_1_-T_2_ dual-mode contrast agent.

**Figure 8:**
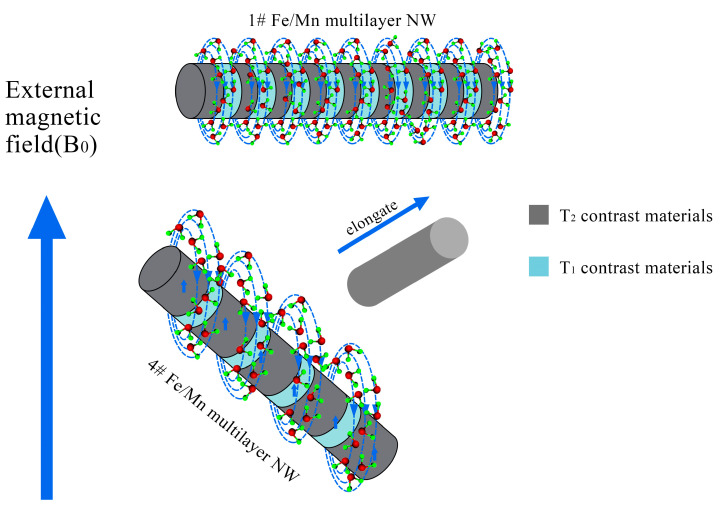
Schematic diagram of the magnetic field model of the multilayer structure of alternate T_1_ and T_2_ materials.

When the length of the Fe layer is different, Fe/Mn@SiO_2_ multilayer NWs show different relaxation. With the decrease in the length of the Fe layer (40 nm~5 nm), the T_2_ relaxation of Fe/Mn@SiO_2_ multilayer NWs first increases gradually (32.1 mM^−1^s^−1^~74.8 mM^−1^s^−1^) and then decreases rapidly (28.9 mM^−1^s^−1^), which may be caused by the differences in the magnetization direction of Fe/Mn@SiO_2_ multilayer NWs with different lengths of the Fe layer. In general, T_2_ relaxation is closely related to such factors as the saturation magnetization of the sample and the number of water molecules covered by a nonuniform magnetic field. The stronger the saturation magnetization provided by the multilayer NWs, or the greater the number of water molecules covered by the magnetic field, the faster the hydrogen protons in the water molecules lose phase, thus accelerating and shortening the T_2_ relaxation process. In the Fe/Mn@SiO_2_ multilayer NWs 1~4# samples prepared by us, the smaller the length–diameter ratio of the Fe layer, the more the easy magnetization direction of the Fe layer tends to be perpendicular to the long axis direction of the NW; on the contrary, the larger the length–diameter ratio is, the easier the magnetization direction tends to be parallel to the NW direction, as shown in Figure 8. When the direction of easy magnetization is perpendicular to the long axis of NWs, the effective area covered by the magnetic field of Fe/Mn@SiO_2_ multilayer NWs is the largest. Therefore, the smaller the aspect ratio of the Fe layer is, the more water molecules are covered by the magnetic field generated by Fe/Mn@SiO_2_ multilayer NWs and the greater the T_2_ relaxation degree of Fe/Mn@SiO_2_ multilayer NWs. Although the shorter the Fe layer is the lower the saturation magnetization of the Fe/Mn@SiO_2_ multilayer NWs is as well, the effective area covered by the magnetic field of the Fe/Mn multilayer NWs plays a leading role in the contribution of T_2_ relaxation. Therefore, the shorter the Fe layer is, the greater the T_2_ relaxation of the Fe/multilayer NWs is.

When the length of the Fe layer is reduced to 5 nm, the size effect of the Fe layer is obvious. The Fe atoms in the Fe layer gather on the surface, which destroys the exchange between Fe atoms. As a result, the saturation magnetization decreases obviously, and T_2_ relaxation decreases sharply. At this time, although the effective area covered by the magnetic field of Fe/Mn@SiO_2_ multilayer NWs is the largest, it still fails to offset the significant influence brought about by the decrease in saturation magnetization, resulting in a dramatic decrease in T_2_ relaxation and the maximum T_2_ relaxation r_2_ of 2# Fe/Mn multilayer NWs. With the decrease in the Fe layer length (40 nm~5 nm), the T_1_ relaxation of the Fe/Mn@SiO_2_ multilayer NWs is slightly affected (21.5 mM^−1^s^−1^~22.3 mM^−1^s^−1^). When the length of the Fe layer decreased to 5 nm, the T_1_ relaxation of the Fe/Mn@SiO_2_ multilayer NWs (24.7 mM^−1^s^−1^) increased slightly. This may be due to the change in magnetic field environment caused by Fe/Mn@SiO_2_ multilayer NWs when the Fe layer length is greater than 5 nm, which has little effect on the precession frequency of hydrogen protons. When the length of the Fe layer is 5 nm, the local magnetic field intensity produced by Fe/Mn@SiO_2_ multilayer NWs makes the precession frequency of hydrogen protons close to that of the surrounding molecules, and the resonance between them accelerates the energy release of high-energy hydrogen protons. Thus, T_1_ relaxation is improved.

Fe/Mn@SiO_2_ multilayer NWs exhibit better T_1_ and imaging effects than Mn single component NWs. This may be due to the high magnetic sensitivity of the Fe layer, which induces the local magnetic field, so that the adjacent paramagnetic Mn layer is enhanced by the induced magnetic field generated by the surrounding Fe layer, thus accelerating the longitudinal relaxation rate of the surrounding water protons. Therefore, Fe/Mn@SiO_2_ multilayer NWs can significantly shorten the longitudinal relaxation time T_1_, then enhance the positive contrast effect of the Fe/Mn@SiO_2_ multilayer NWs. Because the Mn layer length is only 5 nm, the proportion of Mn atoms located on the surface or interface of the Mn layer is larger than that inside the layer, and the ratio increases with the decrease of the Mn layer size. The frequency of the symmetric electrons in Mn is close to that of the water particles. The relaxation rate of the high energy proton spin nucleus is more coordinated with that of the nearby proton spin relaxation through the formation of the electron–proton dipole interaction. The synergistic effect of these two enhancement mechanisms makes Fe/Mn@SiO_2_ multilayer NWs exhibit better coverage and imaging effects. Compared with the Fe NWs, 2# Fe/Mn@SiO_2_ multilayer NWs exhibit better T_2_ relaxation, which may be due to 2# Fe/Mn@SiO_2_ multilayer NWs providing more effective magnetic fields for hydrogen protons.

Although the saturation magnetization (32.5 emu/g) of 2# Fe/Mn@SiO_2_ multilayer NWs is less than that of Fe NWs (48.3 emu/g), at this time, the increase in the effective magnetic field plays a leading role in the contribution of T_2_ relaxation, which counteracts the effect of the decrease of saturation magnetization; in addition, the T_2_ shortening effect from the Mn layer and the synergistic T_2_ shortening effect produced by Mn near the Fe layer further promote the T_2_ relaxation and imaging effects of 2# Fe/Mn@SiO_2_ multilayer NWs.

## 4. Conclusions

- We have successfully developed Fe/Mn multilayer NWs with adjustable Fe layer thickness on the AAO template using the controlled potential double pulse method. The Fe layer is mainly composed of the Fe of bcc structure, and the Mn layer is composed of the Mn of bcc structure, and the Fe3Mn7 is composed of fcc structure.
- The SiO_2_-coated Fe/Mn multilayer NWs have obvious core-shell structure. The thickness of the SiO_2_ layer is uniform and has good dispersion in water. When the Fe layer length is short (≤10 nm), Fe/Mn multilayer NWs exhibit superparamagnetic characteristics.
- MRI studies showed that, when the Fe layer length was about 10 nm and the Mn layer length was about 5 nm, the r_1_ value (21.8 mM^−1^s^−1^) and r_2_ value (74.8 mM^−1^s^−1^) were higher than the r_1_ value of Mn@SiO_2_ NWs (3.7 mM^−1^s^−1^) and the r_2_ value of Fe@SiO_2_ NWs (59.3 mM^−1^s^−1^). This indicates that the Fe/Mn@SiO_2_ multilayer NWs show obvious T_1_ positive enhancement, T_2_ negative enhancement effect, and a good T_1_-T_2_ dual-mode imaging effect.

## Figures and Tables

**Table 1:** Preparation parameters of Fe/Mn multilayer NWs arrays: Fe deposition voltage and time (V1, t1); Mn deposition voltage and time (V2, t2); number of repetitions (N).

Sample	t1 (s)	t2 (s)	V1 (V)	V2 (V)	N
1#	3	5	−1.2	−1.9	50
2#	5	5	−1.2	−1.9	34
3#	8	5	−1.2	−1.9	20
4#	12	5	−1.2	−1.9	11

**Table 2:** The content of Mn and Fe in the Mn layer.

Elements	ms %	mol %
Mn	12.78	87.41
Fe	87.22	12.59

**Table 3:** Size parameters of 1#–4# Fe/Mn multilayer NWs: length of Fe layer (L_Fe_); length of Mn layer (L_Mn_); diameter of nanowires (D); total length of nanowires (L).

Sample	L_Fe_ (nm)	L_Mn_ (nm)	D (nm)	L (μm)
1#	5	5	32.5	0.568
2#	10	5	31.3	0.516
3#	20	5	30.1	0.492
4#	40	5	30.8	0.470

**Table 4:** Magnetic properties of NWs.

Magnetic Properties\NWs	1# Fe/Mn	2# Fe/Mn	3# Fe/Mn	4# Fe/Mn	Fe
Ms (emu/g)	11.3	28.1	38.6	43.2	48.1
Mr (emu/g)	0.1	0.2	3.9	12.2	25.1
Hc (Oe)	8.2	10.3	68.7	81.4	658

**Table 5:** Relaxation of NWs.

NWs	Length (μm)	r_1_ (mM^−1^s^−1^)	r_2_ (mM^−1^s^−1^)	r_2_/r_1_
1# Fe/Mn	0.568	24.7	28.9	1.2
2# Fe/Mn	0.516	22.8	74.8	3.3
3# Fe/Mn	0.492	22.3	55.3	2.5
4# Fe/Mn	0.470	21.5	32.1	1.5
Fe	0.620	2.8	59.3	21.2
Mn	0.681	5.8	11.1	1.9

## Data Availability

Data sharing not applicable.
